# Challenges in Typification Within Taxonomically Complex Groups: The Case of the Linnaean Name *Centaurea phrygia* (Asteraceae)

**DOI:** 10.3390/plants14091336

**Published:** 2025-04-29

**Authors:** Gianmarco Tavilla, Manuel B. Crespo, Pedro Pablo Ferrer-Gallego

**Affiliations:** 1 Independent Researcher, 98121 Messina, Italy; 2 dCARN (Departamento de Ciencias Ambientales y Recursos Naturales), Universidad de Alicante, P.O. Box 99, ES-03080 Alicante, Spain; crespo@ua.es; 3 Servicio de Vida Silvestre y Red Natura 2000, Centro para la Investigación y Experimentación Forestal, Generalitat Valenciana, Avda. Comarques del País Valencià 114, ES-46930 Quart de Poblet, Spain; flora.cief@gva.es

**Keywords:** burser herbarium, *Centaurea* subg. *Centaurea*, *Centaurea* sect. *Phrygia*, compositae, epitype, lectotype, nomenclature

## Abstract

The Linnaean names have undergone significant changes over time, mainly in taxonomically complex aggregates where hybridisation or genetic introgression are frequent. A notable example is the name *Centaurea phrygia*, which Linnaeus applied in 1753 in a broad sense, including several taxa that are now recognised as distinct at the specific or subspecific rank. In this context, the typification of the Linnaean name *C. phrygia* is discussed. The original elements in the protologue, comprising five specimens and one illustration, are critically analysed. The five specimens are excluded for selection as lectotype because they do not correspond to the current concept, use and circumscription of the name *C. phrygia* subsp. *phrygia*. The name is lectotypified using an illustration published by Clusius in his “*Rariorum plantarum historia*”. However, the selected lectotype is demonstrably ambiguous, and the name may not be applied to a single currently recognised taxon with certainty. For that reason, *C. phrygia* may be a clear example for a proposal to conserve the name with a conserved type. However, because the illustration cited by Linnaeus in 1753, and here selected as lectotype, is part of the protologue and therefore cannot be in serious conflict with it or be superseded, we propose a solution to conclusively fix the case of that Linnaean name. Accordingly, we propose an epitype from an element that unambigously represents the current concept and use of the name *C. phrygia* subsp. *phrygia*. The epitype selected is a modern and well-preserved specimen kept at PRC (with barcode PRC452350) and with several duplicates.

## 1. Introduction

*Centaurea* L. (Asteraceae) comprises many taxa, mainly distributed across Europe, the Mediterranean region and southwestern Asia [1,2,3,4,5,6,7]. According to POWO [8], there are 770 accepted species within this genus. Among the three subgenera currently recognised within the genus [9], *C.* subg. *Centaurea* is the most diverse, encompassing up to 20 sections. One of these, *Centaurea* sect. *Phrygia* Pers., comprises plants characterised by a chromosome number of *x* = 11 chromosomes, leaves entire to deeply dentate, and bract appendages either long and fimbriate or short ciliate to membranaceous. However, intermediate bract morphotypes are frequently observed due to hybridisation processes between sympatric taxa [10,11,12,13]. Ecologically, taxa within this section usually grow in subalpine and montane meadows and grasslands, often disturbed by mowing, grazing, or avalanches [9]. Their distribution is largely centred in central and eastern Europe, with the highest diversity occurring in the Balkan Peninsula.

*Centaurea phrygia* L. is the type of this section and represents an aggregate of taxonomically complex entities distributed from central to southeastern Europe, including approximately 15–20 taxa at different ranks. Nine of them, exhibiting a broad range of morphological variation, are often treated as subspecies of *C. phrygia* [3,5,8]. However, other authors [14] have recognised only two subspecies within this variability. In a strict sense, the name *C. phrygia* subsp. *phrygia* is currently applied to perennial plants characterised by long branched stems; leaves ovate to broadly ovate-lanceolate, acuminate, green, laxly hairy, entire to dentate on margins, sessile to subpetiolate; and capitula ca. 20 mm in diameter, numerous, solitary on branches, globose, radiate, mostly covered with appendages of bracts which are provided with 12–20 cilia on each side and gradually taper into a subulate acumen recurved outwards, those of the inner bract distinctly exceeding the middle ones [3]. The species is distributed through northern, eastern, and central Europe, occurring in the northern half of the European Russia, southern Finland, Belarus, Estonia, Latvia, Lithuania, Poland, eastern Germany, Czech Republic, Slovakia, Ukraine, Romania, Bulgaria and Albania. It is also recorded as an adventive species in Denmark, Sweden and Norway [3,5,8,13,15,16].

The occurrence of two dominant cytotypes within *C. phrygia* s.l., diploid (2*n* = 22) and tetraploid (2*n* = 44), along with additional triploid, pentaploid, and hexaploid levels, has been confirmed by Koutecký et al. [13] in populations from Central Europe. Only a diploid chromosome count is known across most of the species distribution range. In contrast, tetraploids appear to have a more restricted distribution, being confined to the Czech Republic and Slovakia [10,17,18,19]. A clear correlation between the two main cytotypes and several morphological traits such as the width of involucre, the length and colour of appendages on the involucral bracts, and the length of pappus, has been reported by Koutecký et al. [13]. Based on these findings, the authors have proposed applying the name *C. phrygia* s.str. to diploid populations, while recognising tetraploid populations under the name *C. erdneri* J.Wagner (pro hybr.) (for further details, see Koutecký et al. [13]).

The taxonomy of *C.* sect. *Phrygia* is quite complex. Taxonomic differentiation has traditionally been based on leaf features, the size and shape of bract appendages, and the relative length of pappus [3,13,20]. However, leaf morphology (including size, shape and margin division), as well as indumentum, can vary considerably among populations of a single species. In this scenario, the typification of old names, for which precise localities are often lacking, presents a significant challenge. This difficulty arises not only from the intrinsic taxonomic complexity of the group but also from the frequent heterogeneity of the original material.

To the best of our knowledge, the name *Centaurea phrygia* has not yet been typified ([13,21]; see also http://www.nhm.ac.uk/our-science/data/linnaean-typification/, accessed on 5 February 2025). A previous reference to a type by Tzvelev (in Klokov et al. [22] (p. 451)) in *Flora URSS*, as “Type in London”, should not be considered as an effective lectotypification of the name (Art. 9.12 of the *ICN*; Turland et al. [23]).

In light of the aforementioned context and as part of our ongoing nomenclatural study of names in *Centaurea* [24,25,26,27,28,29,30,31,32], we here undertake the lectotypification of *C. phrygia*, a name referring to a taxonomically complex group that includes several ploidy levels. A lectotype and an epitype are designated below to maintain the current usage of this name for diploid plants within the complex, predominantly distributed across central and northern Europe.

## 2. Results and Discussion

Linnaeus’s protologue of *Centaurea phrygia* consists of a diagnosis “CENTAUREA calycibus recurvato-plumosis, foliis indivisis”, followed by four synonyms: (1) “Centaurea calycibus ciliatis: ciliis setaceis recurvatis” cited from Linnaeus ([33] (p. 422); [34] (p. 270)), Van Royen ([35] (p. 139)) and Dalibard ([36] (p. 266)), (2) “Jacea latifolia & angustifolia, capite hirsuto” cited from Bauhin ([37] (p. 271)), (3) “Jacea IIII austriaca, capite villoso” cited from Clusius ([38] (p. 7 vii)), and (4) the varietal name “β Jacea alba, hirsuto capite” from Bauhin ([37] (p. 271)). The protologue also includes the provenance “*Habitat in* Helvetiae, Austriae, Finlandia”, and the symbol “
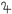
” indicating a perennial plant. Clusius ([38]: 7 vii) provided an illustration that can be considered original material used by Linnaeus to describe *C. phrygia* (see below).

**Figure 1:**
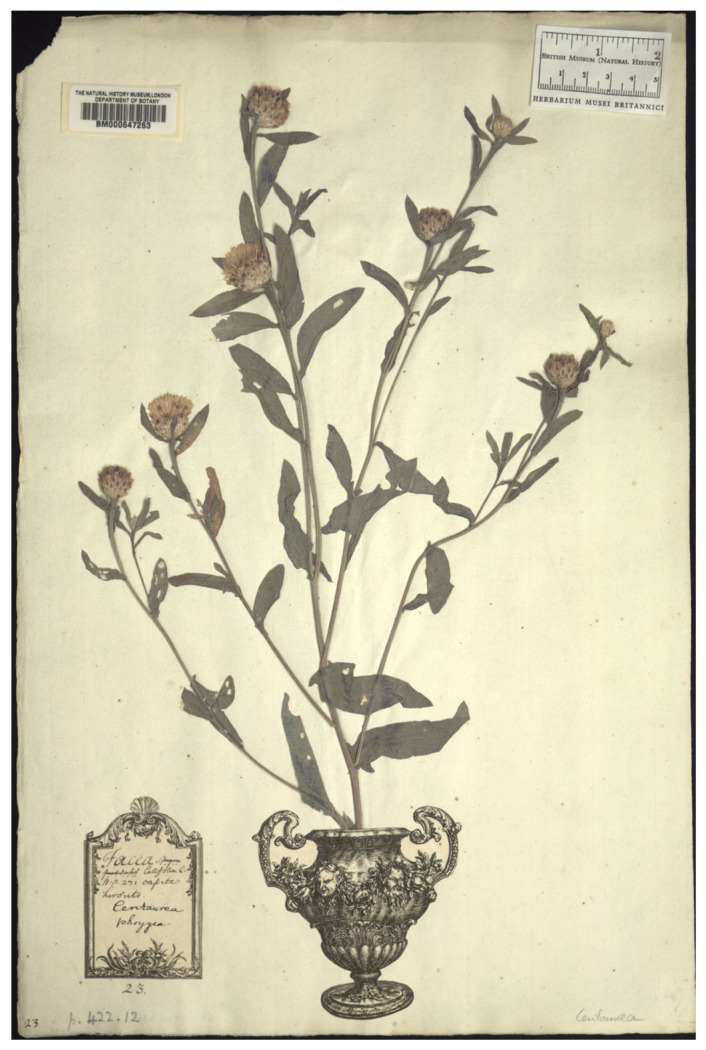
Original material of *Centaurea phrygia* L., BM barcode BM000647263 (Image by courtesy of the herbarium BM, reproduced with permission).

Among the original material cited by Jarvis ([21] (p. 401)), five specimens are preserved at UPS, BM and LINN herbaria. In the Clifford Herbarium at BM, a single sheet (barcode BM000647263) contains a well-preserved and fully developed specimen that corresponds to Linnaeus’s protologue and qualifies as original material for *C. phrygia* (Figure 1). Moreover, Clifford’s annotation “Jacea nigra pratensis latifolia C. B. S. 271 capite hirsuto” establishes a link to one of the Bauhin’s synonyms. The specimen was apparently cultivated in the Netherlands from an unknown source. However, the plant fragment on this sheet does not conform to the current concept of *C. phrygia* subsp. *phrygia* and should therefore be excluded as a potential lectotype. It appears instead to belong to the *C. jacea* L. aggregate.

The Linnaean Herbarium at LINN houses a sheet (Herb. Linn. No. 1030.7; image available at: http://linnean-online.org/10612/, accessed on 5 February 2025), which constitutes original material for the name. This specimen bears Linnaeus’s annotation “7 *phrygia*”, where “7” explicitly corresponds to the species number of *C. phrygia* in *Species Plantarum* [39]. As noted by Jarvis [21], the presence of the 1753 *Species Plantarum* number on a sheet in the Linnaean Herbarium in London is taken as evidence that the specimen was in Linnaeus’s possession at the time, thereby confirming its status as original material. Additionally, the sheet includes the abbreviation “HU”, indicating that the plant was cultivated in the Uppsala botanical garden “Hortus Upsaliensis”. The specimen largely conforms to the current concept of *C. phrygia* subsp. *phrygia*, although certain characteristics of the capitula and bracts exhibit slight deviations, possibly aligning more closely with *C. pseudophrygia* C.A.Mey. Nevertheless, despite the absence of clear provenance data, this specimen could be considered a potential candidate for lectotypification.

In the Joachim Burser Herbarium at UPS-BURSER, three herbarium sheets are relevant for the typification of the name: Herb. Burser XV[2]: 30, 31 & 32 (UPS-BURSER). Usually, Linnaeus’s citation of the polynomial from Bauhin’s *Pinax* [37] establishes an indirect link to the specimens of Burser’s herbarium at UPS-BURSER. The Joachim Burser Herbarium (UPS-BURSER) was arranged and labelled according to Caspar Bauhin’s *Pinax* [37]. This herbarium was used by Linnaeus for the interpretation of the names that appear in Bauhin’s work.

**Figure 2:**
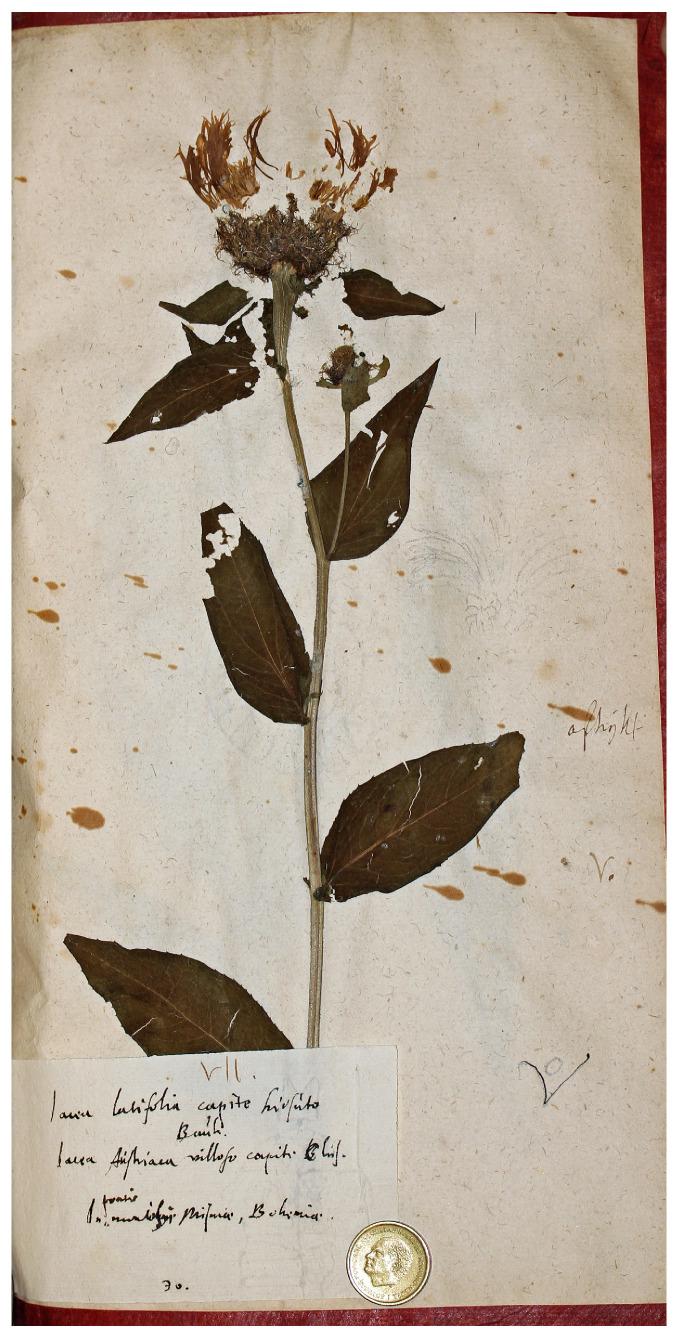
Original material of *Centaurea phrygia* L., Herb. Burser XV[2]: 30 (Image by courtesy of the herbarium UPS-BURSER, reproduced with permission).

The sheet Herb. Burser XV[2]: 30 (No. V-174704) is labelled “Iacea latifolia capite hirsuto Bauh./Iacea austriaca capite villoso Clus/In pratis montibus Misniae, Bohemiae”, and contains a poorly preserved fragment, a single well-developed flowering head (Figure 2). The specimen was collected in the surroundings of Meissen, in eastern Germany, near the Czech border. While this fragment matches the current concept of *C. phrygia* subsp. *phrygia* (or *C. pseudophrygia*), the only well-preserved capitulum is broader than expected, possibly indicating introgression with other members of the genus. This, combined with extensive insect damage, suggests that it should be set aside from consideration for lectotypification.

**Figure 3:**
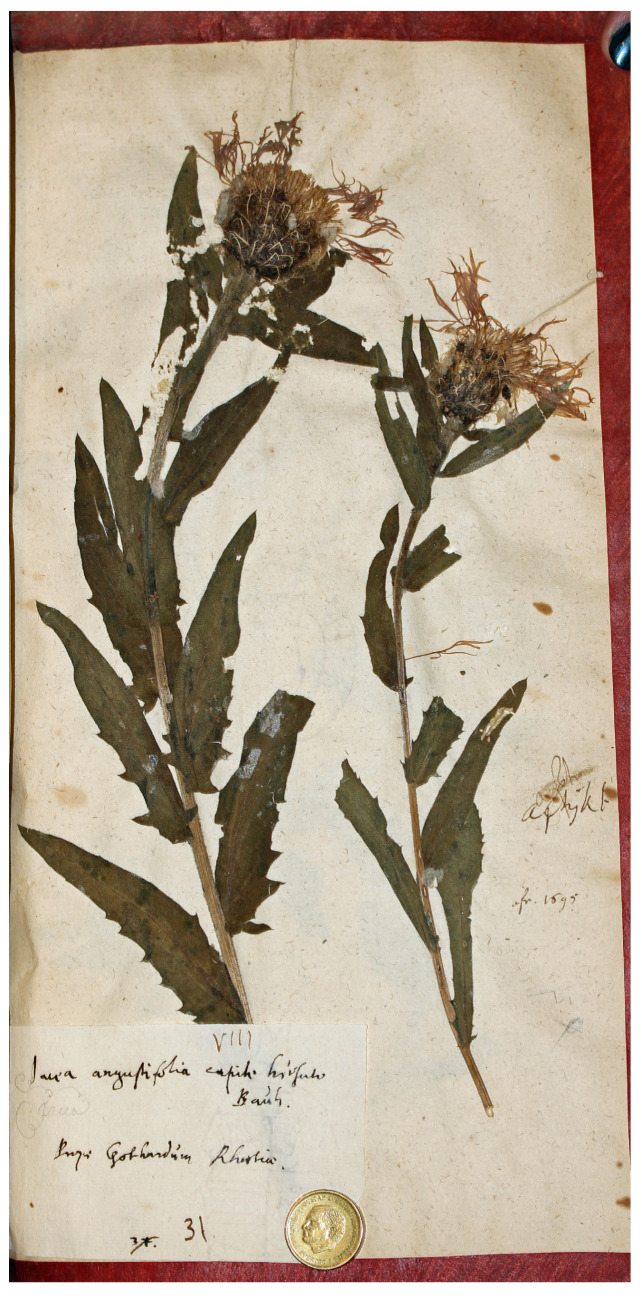
Original material of *Centaurea phrygia* L., Herb. Burser XV[2]: 31 (Image by courtesy of the herbarium UPS-BURSER, reproduced with permission).

The sheet Herb. Burser XV[2]: 31 (No. V-174705) contains two fragments of the same species, each with leaves and a single flowering head (Figure 3). It is labelled “Iacea angustifolia capite hirsuto Bauh/Prope Gothardum Rhaetiae”, which matches the Linnaean synonym from Bauhin [37]. The specimen was probably collected near Saint Gotthard Pass, in the Lepontine Alps of southern Switzerland, close to the Italian border. This material matches the current concept of *C. nervosa* and should therefore be excluded from consideration as a potential lectotype for *C. phrygia*.

**Figure 4:**
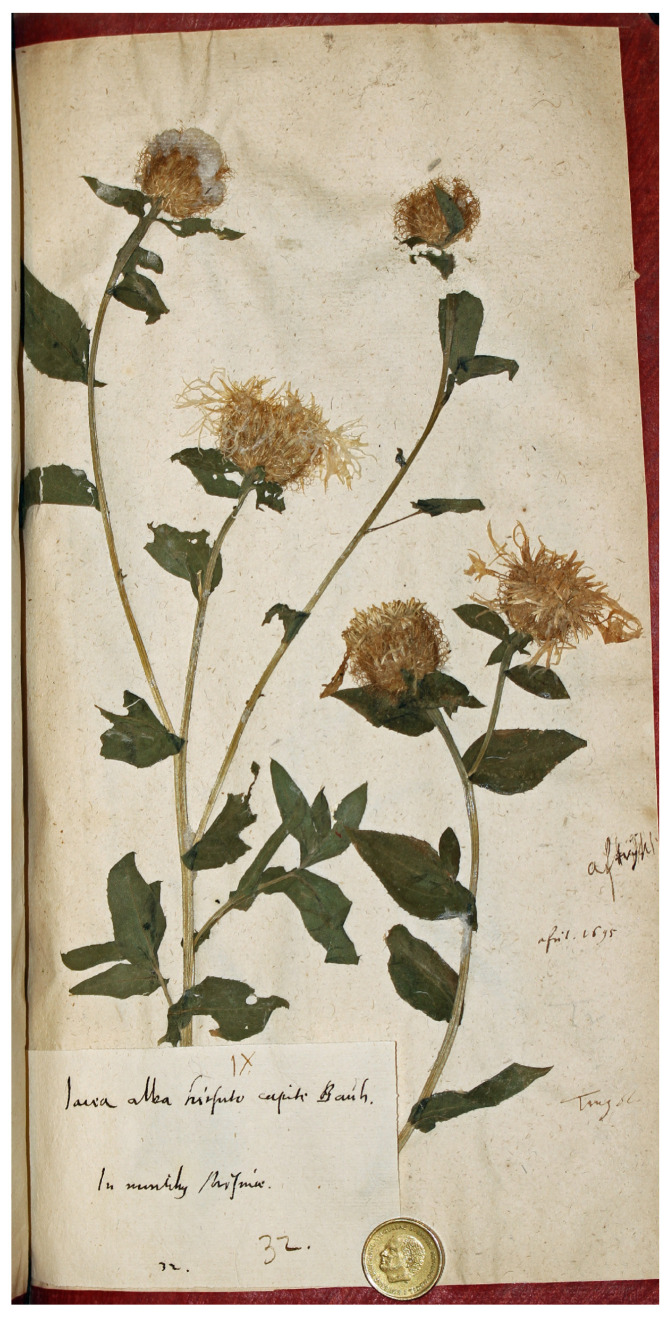
Original material of *Centaurea phrygia* L., Herb. Burser XV[2]: 32 (Image by courtesy of the herbarium UPS-BURSER, reproduced with permission).

Finally, the sheet Herb. Burser XV[2]: 32 (No. V-174706), preserved at UPS, contains a plant fragment accompanied by a label with the annotation “Iacea alba hirsuto capite Bauh/In montibus Misniae [Bohemia]/32”, linking it to the unnamed variety β (Figure 4). This material was also gathered near Meissen (eastern Germany) and matches the current concept of *C. phrygia* subsp. *phrygia*, although certain features of the capitula and bract appendages suggest affinities with *C. pseudophrygia*. While the specimen is exceptionally well preserved, it may not be the most suitable choice for lectotypification.

**Figure 5:**
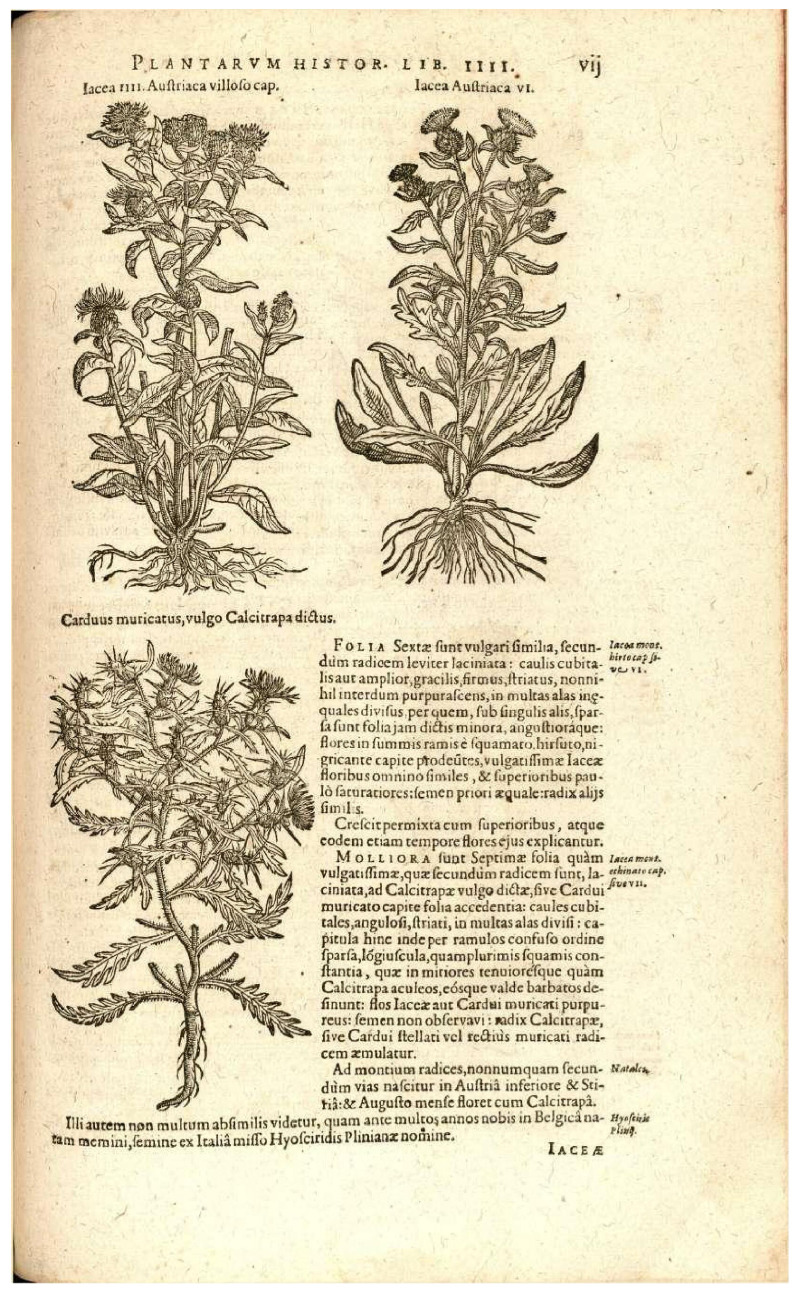
Lectotype of *Centaurea phrygia* L., illustration “Iacea IIII Austriaca villoso cap. [capite]” in Clusius (1601: vii), upper left plant.

Furthermore, Clusius ([38] (p. vii)) provided an illustration of his “Iacea IIII Austriaca villoso capite”, explicitly cited by Linnaeus in the protologue (Figure 5). As part of the original material, this illustration is valuable for typification purposes. It depicts an Austrian or Hungarian plant described as “Iacea montana villoso capite, sive IIII. elatior”, reportedly collected “in montanis Pannoniae & Austriae pratis”. However, its precise identity remains uncertain, as it could correspond to *C. phrygia*, *C. pseudophrygia* (*C. austriaca* auct., non Willd.) or *C. stenolepis* A.Kern. In cases where the original herbarium material is highly heterogeneous, and particularly when it does not align with the current concept or usage of the name, iconography can provide a solution to fix the concept of a name, especially in the absence of syntypes, and thus without the need to comply with Article 9.12 of the *ICN*. The illustrations cited in the protologue may serve as an alternative to proposing the conservation of the name with a conserved type. Based on the articles of the *ICN*, this can be achieved through lectotypification based on the iconography (an illustration cited in the protologue is part of the protologue and therefore cannot be in serious conflict with it or be superseded); and, if necessary, the lectotype designated can be supported by the designation of an epitype, as we justify below for this Linnaean name.

Two additional herbarium sheets containing specimens of this species are preserved in the Linnaean herbarium at the Swedish Museum of Natural History (S). The sheet at S, with code S09-20344 (image available at https://herbarium.nrm.se/specimens/S09-20344, accessed on 5 February 2025), is annotated “*Centaurea phrygia* Linn”. by J.E. Wikström and contains only a plant fragment with capitula and leaves, which closely matches both the protologue and the current concept of *C. phrygia* s.l. The sheet bears the annotation “7 *phrygia*” at its base (the species number of *C. phrygia* in Linnaeus’s *Species Plantarum*), though this was likely not written by Linnaeus himself. Additional annotations on the reverse side of the sheet include “Herb. Alstroemeri”, and “a Linné f.” by A. Dahl. As this specimen was probably added to the collection after 1753, it cannot be considered original material for the name [see Jarvis [21] (p. 401)].

The second sheet at S, with the code S09-20306 (image available at https://herbarium.nrm.se/specimens/S09-20306, accessed on 5 February 2025), bears a fragment that corresponds to both the protologue and the current concept of *C. phrygia* s.l.. The sheet is annotated “*Centaurea phrygia* L.”, though not in Linnaeus’s handwriting, and was likely inscribed by L.J. Montin. Additionally, the numbers “7” and “spec. pl. 910” are written in pencil, but these represent later additions to the sheet, made after the publication of *Species Plantarum*, and thus render it ineligible as type. On the reverse side of the sheet, the annotations include “Herb. Montin” and a description reading “Centaurea (phrygia) calycibus recurvato-plumosis, foliis indivisis, oblongis glabris. Syst. nat. ed. 13, p. 8”. The sheet also bears the geographical locality cited in the protologue, Linnaeus’s symbol for a “perennial plant” (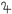), and the note “Specimen ex horto Upsal. communicavit Hortulanus Nietzel” [Dietrich Nietzel], indicating that the specimen was provided from the Uppsala garden.

We have been unable to trace any further original material of *C. phrygia* in any of the other Linnaean and Linnaean-linked herbaria, including L, where any potential specimen could have been associated with the synonym cited by Van Royen [35] in the protologue.

Based on the synonyms and type localities inferred from the protologue, the name *C. phrygia,* as conceived by Linnaeus (l.c.), includes an aggregate of morphologically similar entities, which can now be assigned to at least three different taxa: *C. pseudophrygia* (*C. phrygia* subsp. *pseudophrygia* (C.A.Mey.) Gugler), *C. nervosa* Willd., and the true *C. phrygia* s.str. These entities are mostly allopatric and can be distinguished by several morphological and cytological features (cf. Koutecký [10]; Koutecký et al. [13]). However, in extensive areas of central Europe, some of these taxa co-occur, leading to hybridisation among different members of *C.* sect. *Phrygia* (cf. Koutecký [40]; Koutecký et al. [13]; López-Alvarado et al. [41]). This taxonomic complexity greatly complicates species and subspecies delimitation and presents significant challenges for the typification of historical names, which are often based on incomplete specimens and/or illustrations. The characteristics of capitula and bract appendages are reliable for taxon identification within *C.* sect. *Phrygia*, and they may represent the only consistent criteria for identification of old materials, since specimens often lack precise locality data.

Among the original material of *C. phrygia*, some specimens could be considered for lectotypification. However, certain sheets must be excluded, as they clearly correspond to either *C. nervosa* (Herb. Burser XV[2]: 31) or to members of the *C. jacea* aggregate (BM 000647263). Other specimens traditionally referred to *C. phrygia* (Herb. Burser XV[2]: 30, Herb. Burser XV[2]: 32, and LINN 1030.7) cannot be confidently assigned to the type subspecies, since the specimens are apparently somewhat deviant and could be interpreted as introgressive or intermediate with *C. pseudophrygia*. Consequently, these should be excluded from lectotypification. While this material represents the traditional Linnaean concept of the name, it does not align with the current concept and use of *C. phrygia* subsp. *phrygia*. A lectotypification of the name *C. phrygia* on the plants of these sheets would be nomenclaturally disruptive.

In this context, it seems appropriate to focus on the identity of Clusius’s ([38] (p. vii)) illustration of “Iacea IIII Austriaca villoso capite” to support the continued and well-established use of the name *C. phrygia*. This figure, a complete plant with leaves and inflorescences, was drawn from material collected in an unspecified mountainous region of Austria or Hungary, a vast area where the true *C. phrygia* subsp. *phrygia* (*C. austriaca* Willd.), *C. pseudophrygia* (*C. austriaca* auct., non Willd.) or *C. phrygia* subsp. *stenolepis* (A.Kern.) Gugler (*C. stenolepis* A.Kern.) occur altogether. Koch [42] assigned this illustration to his own interpretation of “*C. phrygia*”, a name that actually corresponds to *C. pseudophrygia,* as later described by Meyer [43].

However, these three taxa can only be reliably distinguished based on the characteristics of their bract appendages and fruits, two diagnostic structures that are not depicted in detail in Clusius’s illustration. Despite this limitation, the illustration still provides a practical and satisfactory basis for typification. If it were selected as the lectotype for *C. phrygia* s.l., the designation of an epitype (Art. 9.9 of the *ICN*) based on a securely identified specimen belonging to the typical subspecies would allow for the continued use of the name in its current sense. In fact, the concept of epitypes was introduced to address issues where the primary type of a plant name (e.g., lectotype) is demonstrably ambiguous and cannot be identified at the appropriate taxonomic level [44].

**Figure 6:**
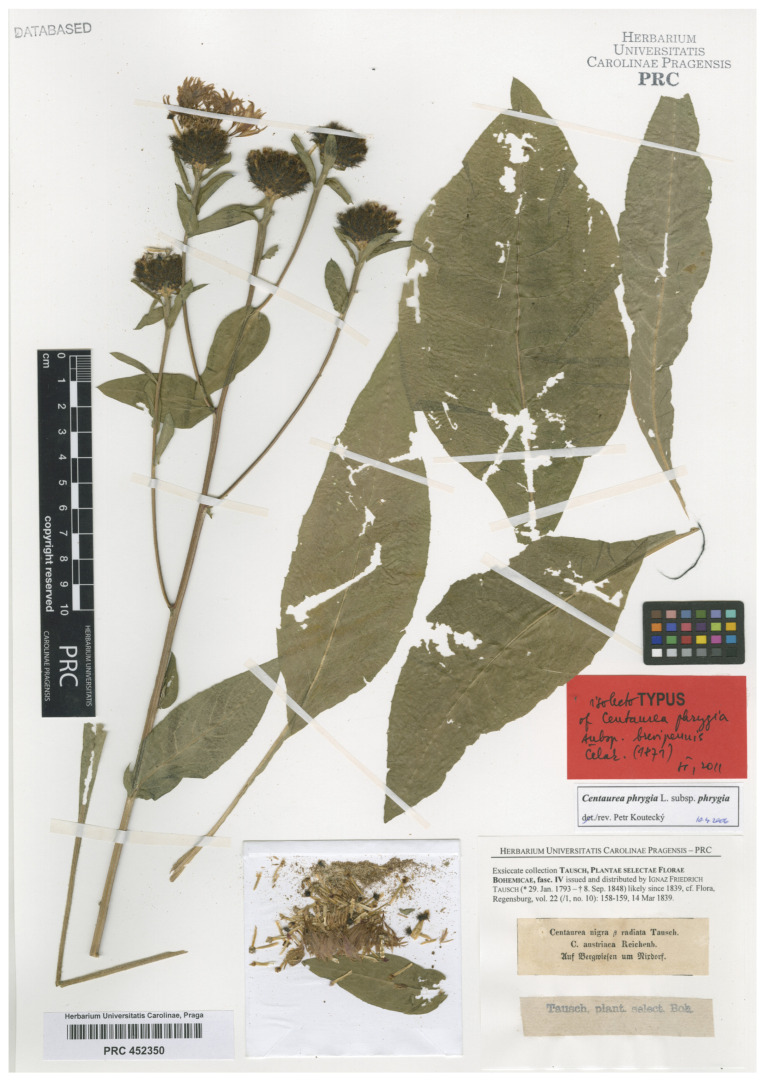
Epitype of the name *Centaurea phrygia* L. subsp. *phrygia*, PRC barcode PRC452350 (Image by courtesy of the herbarium PRC, reproduced with permission).

We consider this a reasonable approach to preserving the traditional usage of that Linnaean name, thereby avoiding the potential scenario in which one of the available specimens is later demonstrated, potentially through molecular analysis, to belong to a different taxon or a nothotaxon. Consequently, for this purpose, the selection of one specimen collected by Tausch of *Centaurea nigra* β *radiata* Tausch. (PRC452350) appears to be the best choice (Figure 6), since this name is widely accepted as *Centaurea phrygia* subsp. *brevipennis* Čelak, which is a synonym of *C. phrygia* subsp. *phrygia*. The sheet contains a well preserved and fully developed plant, previously identified by P. Kouteck as the true specimen of *C. phrygia* subsp. *phrygia,* who later designated the lectotype of C. *phrygia* subsp. *brevipennis* (PRC 452349; Koutecký et al. [13]). The specimen at PRC selected as epitype was collected in Czech Republic on mountain meadows around Nixdorf (=Mikulášovice, Bohemia), where only diploids occur. Moreover, according to Koutecký & al. [13], the name *C. phrygia* subsp. *brevipennis* can be unambiguously attributed to the diploid cytotype, aligning with Linnaeus’s protologue as well as the traditional and current circumscription of *C. phrygia s. str*.

***Centaureaphrygia*** L., Sp. Pl.: 910. 1753 subsp. ***phrygia—*****Lectotype(designated here):** [illustration] “*Iacea IIII Austriaca villoso cap.* [*capite*]” in Clusius, Rariorum plantarum historia: vii. 1601.—**Epitype (designated here):** “*Centaurea nigra* β *radiata Tausch. C. austriaca Reichenb. Auf Bergwiesen um Nixdorf”,* s.d., *sine dato legit I. F. Tausch s.n.* (PRC barcode PRC452350!). Isoepitypes: PRC barcodes PRC452349, PRC452351, and PRC452349, PRC452348.

## 3. Materials and Methods

To trace the original material of the name and for typification purposes the protologue and other relevant publications cited by Linnaeus [39] were thoroughly examined. Research was carried out where the types and specimens used by Linnaeus are typically kept [45]. Five herbarium sheets, housed at BM, LINN, and UPS-BURSER (acronyms following Thiers [46]; continuously updated), along with one illustration considered to be original material [21], were carefully evaluated. Additionally, two further sheets from the Linnaean collection at S were examined to provide a more comprehensive interpretation of the nomenclature issue. The cited articles follow the *Shenzhen Code* (Turland et al. [23], hereafter *ICN*).

## 4. Conclusions

The typification process is crucial for establishing a precise taxonomic identity and ensuring nomenclatural stability. Prior to 1 January 1958, the designation of type specimens was not a mandatory requirement, and it currently concerns many Linnaean names that lack clear type material. Regarding *Centaurea phrygia*, which belongs to a taxonomically complex group within the Asteraceae, lectotypification has been undertaken here on ambiguous original material, followed by epitypification on a recognisable modern material. Both steps were deemed necessary under the *Shenzhen Code* to maintain the current use of the name and will surely provide a stable foundation for further taxonomic studies on this taxon and its relatives. 

## Data Availability

The authors confirm that the data supporting the findings of this study are available within the article.

## References

[1] Wagenitz G., Davis P.H. (1975). Centaurea L.. Flora of Turkey and the East Aegean Islands, vol. 5.

[2] Wagenitz G. (1986). *Centaurea* in South-West Asia: Patterns of distribution and diversity. Proc. R. Soc. Edinb..

[3] Dostál J., Tutin T., Heywood V., Burges N., Valentine D., Walters S., Webb D. (1976). Centaurea L.. Flora Europaea 4.

[4] Hellwig F. (2004). Centaureinae (Asteraceae) in the Mediterranean–history of ecogeographical radiation. Plant Syst. Evol..

[5] Greuter W. Compositae (Pro Parte Majore). https://europlusmed.org/cdm_dataportal/taxon/599e0bdb-a2e8-49b9-9907-5398259c4f00.

[6] Susanna A., Garcia-Jacas N., Kadereit J., Jeffrey C. (2007). Tribe Cardueae. The Families and Genera of Vascular Plants, Flowering Plants. Eudicots. Asterales, vol. VIII.

[7] Susanna A., Garcia-Jacas N., Funk V., Susanna A., Stuessy T., Bayer R. (2009). Tribe Cardueae. Systematics, Evolution, and Biogeography of Compositae.

[8] POWO Centaurea L. in Plants of the World Online. Facilitated by the Royal Botanic Gardens, Kew. https://powo.science.kew.org/taxon/urn:lsid:ipni.org:names:330045-2.

[9] Hilpold A., Garcia-Jacas N., Vilatersana R., Susanna A. (2014). Taxonomical and nomenclatural notes on *Centaurea*: A proposal of classification, a description of new sections and subsections, and a species list of the redefined section *Centaurea*. Collect. Bot..

[10] Koutecký P. (2007). Morphological and ploidy level variation of *Centaurea phrygia* agg. (Asteraceae) in the Czech Republic, Slovakia and Ukraine. Folia Geobot..

[11] Vonica G., Cantor M. (2011). The polymorphism and hybridization of the *Centaurea* species. Bull. UASVM Hortic..

[12] Vonica G., Frink J., Cantor M. (2013). Taxonomic revision of some taxa of Jacea-Lepteranthus group (*Centaurea genus*) based on morphometric analysis. Brukenthal Acta Mus..

[13] Koutecký P., Štěpánek J., Baďurová T. (2012). Differentiation between diploid and tetraploid *Centaurea* phrygia: Mating barriers, morphology, and geographic distribution. Preslia.

[14] Hultén E., Fries M. (1986). Atlas of North European Vascular Plants North of the Tropic of Cancer.

[15] Meusel H., Jäger E. (1992). Arealtypen der süd-mitteleuropäischen Flora. Vgl. Chorologie ZentraleuropäIschen Flora.

[16] Alm T., Piirainen M., Often A. (2009). *Centaurea phrygia* subsp. *phrygia* as a German polemochore in Sør-Varanger, NE Norway, with notes on other taxa of similar origin. Bot. Jahrb. Syst. Pflanzengesch. Pflanzengeogr..

[17] Majovsky J., Murin A. (1987). Karyotaxonomicky prehľad Flory Slovenska [Karyotaxonomic Survey of the Flora of Slovakia].

[18] Dostál J. (1989). Nova Květena Československe Socialisticke Republiky [New Flora of the Czechoslovak Socialistic Republic].

[19] Štěpanek J., Koutecký P., Slavík B., Štěpánková J. (2004). Centaurea L.. Květena Česke Republiky [Flora of the Czech Republic].

[20] Arnelas I., Devesa J. (2011). Revisión taxonómica de *Centaurea* sect. Jacea (Mill.) Pers. (Asteraceae) en la Península Ibérica. Acta Bot. Malacit..

[21] Jarvis C. (2007). Order out of Chaos. Linnaean Plant Names and Their Types.

[22] Klokov M., Sosnowsky D., Tzvelev N., Czerepanov S. (1963). Centaurea.

[23] Turland N., Wiersema J., Barrie F., Greuter W., Hawksworth D., Herendeen P., Knapp S., Kusber W.H., Li D.Z., Marhold K. (2018). International Code of Nomenclature for algae, fungi, and plants (Shenzhen Code) adopted by the Nineteenth International Botanical Congress Shenzhen, China, July 2017; Regnum Vegetabile 159.

[24] Ferrer-Gallego P., Altınordu F. (2016). Typification of four Linnaean names in *Centaurea* (Asteraceae). Ann. Bot. Fenn..

[25] Altınordu F. (2016). Typification of the Linnaean name *Centaurea sibirica* (Asteraceae). Phytotaxa.

[26] Altınordu F., Ferrer-Gallego P. (2016). Typifications of Linnaean names in the genus *Centaurea* and *Serratula* (Asteraceae). Nord. J. Bot..

[27] Altınordu F., Ferrer-Gallego P. (2016). Typifications of the Linnaean names *Centaurea eriophora* and *C. orientalis* (Asteraceae). Phytotaxa.

[28] Altınordu F., Ferrer-Gallego P. (2015). Typification of the Linnaean name Centaurea crocodylium (Asteraceae). Phytotaxa.

[29] Tavilla G., Lanfranco S. (2024). Neotypification of the name *Centaurea crassifolia* Bertol. (Asteraceae). Adansonia.

[30] Ferrer-Gallego P., Roselló R., Laguna E., Gómez J., Peris J. (2014). Typification of two Linnaean names: *Centaurea aspera* and *Centaurea isnardii* (Asteraceae). Phytotaxa.

[31] Ferrer-Gallego P., Roselló R., Laguna E., Guill A., Gómez J., Peris J. (2014). Typification of the Linnaean name *Centaurea seridis* (Asteraceae). Phytotaxa.

[32] Ferrer-Gallego P., Susanna A., Laguna E., Guara M. (2014). Lectotypification of Centaurea alpina L. (Compositae) and the identity of Centaurea linaresii Lázaro Ibiza. Taxon.

[33] Linnaeus C. (1738). Hortus Cliffortianus.

[34] Linnaeus C. (1748). Hortus Upsaliensis.

[35] van Royen A. (1740). Florae Leydensis Prodromus, Exhibens Plantas Quae in Horto acadéMico Lugduno-Batavo Aluntur.

[36] Dalibard M. (1749). Florae Parisiensis Prodromus.

[37] Bauhin C. (1623). Pinax Theatri Botanici.

[38] Clusius C. (1601). Rariorum Plantarum Historia.

[39] Linnaeus C. (1753). Species Plantarum.

[40] Koutecký P. (2009). Taxonomic and nomenclatural revision of *Centaurea subjacea* (Asteraceae-Cardueae) and similar taxa. Phyton Ann. Rei Bot..

[41] López-Alvarado J., Sáez L., Filigheddu R., Garcia-Jacas N., Susanna A. (2014). The limitations of molecular markers in phylogenetic reconstruction: The case of *Centaurea* sect. Phrygia (Compositae). Taxon.

[42] Koch W. (1844). Synopsis florae Germanicae et Helveticae.

[43] Meyer C. (1848). Ein paar Worte über *Centaurea phrygia* Linn. Bull. Acad. Imp. Sci. St.-Pétersb..

[44] Sennikov A.N. (2022). The concept of epitypes in theory and practice. Nord. J. Bot..

[45] HUH Botanist Search-HUH-Databases-Harvard University. https://kiki.huh.harvard.edu/databases/botanist_search.php?mode=details&id=92.

[46] Thiers B.M. Index Herbariorum: A Global Directory of Public Herbaria and Associated Staff. http://sweetgum.nybg.org/science/ih/.

